# Lesion Material From *Treponema*-Associated Hoof Disease of Wild Elk Induces Disease Pathology in the Sheep Digital Dermatitis Model

**DOI:** 10.3389/fvets.2021.782149

**Published:** 2022-01-12

**Authors:** Jennifer H. Wilson-Welder, Kristin Mansfield, Sushan Han, Darrell O. Bayles, David P. Alt, Steven C. Olsen

**Affiliations:** ^1^Infectious Bacterial Diseases of Livestock Research Unit, National Animal Disease Center, Agricultural Research Service, United States Department of Agriculture (USDA), Ames, IA, United States; ^2^Washington Department of Fish and Wildlife, Spokane Valley, WA, United States; ^3^Colorado State University Diagnostic Medicine Center, Fort Collins, CO, United States

**Keywords:** *Treponema*, hoof disease, disease transmission, model, wildlife, elk, sheep, *Cervus elaphus*

## Abstract

A hoof disease among wild elk (*Cervus elaphus*) in the western United States has been reported since 2008. Now present in Washington, Oregon, Idaho, and California, this hoof disease continues to spread among elk herds suggesting an infectious etiology. Causing severe lesions at the hoof-skin junction, lesions can penetrate the hoof-horn structure causing severe lameness, misshapen hooves, and in some cases, sloughed hooves leaving the elk prone to infection, malnutrition, and premature death. Isolated to the feet, this disease has been termed treponeme-associated hoof disease due to the numerous *Treponema* spp. found within lesions. In addition to the *Treponema* spp., treponeme-associated hoof disease shares many similarities with digital dermatitis of cattle and livestock including association with several groups of anaerobic bacteria such as Bacteroides, Clostridia, and Fusobacterium, neutrophilic inflammatory infiltrate, and restriction of the disease to the foot and hoof tissues. To determine if there was a transmissible infectious component to this disease syndrome, elk lesion homogenate was used in a sheep model of digital dermatitis. Ten animals were inoculated with lesion material and lesion development was followed over 7 weeks. Most inoculated feet developed moderate to severe lesions at 2- or 4-weeks post-inoculation timepoints, with 16 of 18 feet at 4 weeks also had spirochetes associated within the lesions. Histopathology demonstrated spirochetes at the invading edge of the lesions along with other hallmarks of elk hoof disease, neutrophilic inflammatory infiltrates, and keratinocyte erosion. *Treponema*-specific PCR demonstrated three phylotypes associated with elk hoof disease and digital dermatitis were present. Serum of infected sheep had increased anti-*Treponema* IgG when compared to negative control sheep and pre-exposure samples. Analysis of the bacterial microbiome by sequencing of the bacterial 16S rRNA gene showed a community structure in sheep lesions that was highly similar to the elk lesion homogenate used as inoculum. Bacteroidies, Fusobacterium, and Clostridia were among the bacterial taxa overrepresented in infected samples as compared to negative control samples. In conclusion, there is a highly transmissible, infectious bacterial component to elk treponeme-associated hoof disease which includes several species of *Treponema* as well as other bacteria previously associated with digital dermatitis.

## Introduction

In 2008 increased numbers of limping elk (*Cervus elaphus*) and an increased presence of deformed hoofs were observed in southwestern Washington State. Throughout the following decade, cases were confirmed all along the western range of the Cascade mountains, further north into Washington, throughout neighboring Oregon state, and as far south as northern California. Recently, cases have been confirmed in far eastern Washington state, and adjacent Idaho, representing movement of this disease across to the eastern side of the Cascade mountains. Animals presented as severely lame, often with overgrown, misshaped, or completely sloughed hooves. Reports rapidly increased over a short period of time radiating from the region where lesions were first observed (1). Preliminary investigations indicated an infectious agent as the etiology as other systemic, metabolic, or environmental causes could not be identified (1). Bacterial species isolated from the hoof lesions were similar to those present in polymicrobial digital dermatitis (DD) of livestock (2, 3). Histologic examination of affected tissues revealed spirochete bacteria at the leading/invading edge of the lesions. *Treponema* isolates from elk hoof lesions were determined to be the same three phylotypes as isolated from DD lesions of cattle and sheep (2, 4).

The disease spread outward rapidly from a single area of origin, but there was a lack of other etiologic factors. The current hypothesis is that this elk hoof disease, called Treponeme-associated hoof disease (TAHD), is a transmissible bacterial disease, and most likely poly-bacterial (2, 3). Koch's postulates, greatly simplified, would be that utilization of a disease-causing organism in healthy naïve hosts will reproduce similar clinical signs and subsequently allow re-isolation of the infectious agent from lesions. The Bradford Hill criteria, more widely used in epidemiology fields, contains five criteria for examining a disease and causation: (1) strength of association, (2) consistency, (3) specificity, (4) temporality, and (5) plausibility (5). In both TAHD and DD of livestock, several bacteria have been suspected to be included in the disease pathogenesis, but demonstration of a precise etiology remains elusive. To date, several species of *Treponema* and other anaerobic bacteria are consistently associated with DD in livestock lesions (6). Other variables, such as wet hoof environment, seasonality, high stocking rates or population density, and nutritional or metabolic stress brought on by parturition, early lactation, or poor forage contribute to susceptibility of cattle to DD, small ruminants to contagious ovine digital dermatitis, or elk to TAHD (1, 3, 7–10). The ability to model or reproduce all cofactors in an animal model may not be necessary to fulfill or demonstrate causality of an etiologic factor to onset of disease.

To study hoof diseases in controlled settings, various models of naturally occurring hoof diseases have been used. Most involve housing animals with wet bedding, using boots, bandages, or a system of wraps to macerate the skin and hold bacterial cultures or lesion material in place (11–15). Recently, cell-based models have even been proposed but appear to be limited to evaluating pathogenesis of a single bacterial species at a time (16, 17). Models for DD of cattle have been developed using lengthy macerations (12) or initial abrasion of the skin to provide bacterial inoculum for a site for penetration and lesion production (13, 15). All models require extensive handling of the animal and their feet. However, under research settings the safety of care staff and animals must be considered.

Large cervids, including elk (*Cervus elaphus*) and white-tailed deer (*Odocoileus virginianus*), can be raised in captivity and have been used in infectious disease research (18–22). Although stress and injury can occur in animals not acclimated to human interaction, confined spaces, or handling facilities (23), cervids, even captive bred animals, appear especially susceptible. Injury remains a leading cause of mortality among captive cervids (24–26). Large animals also pose an injury risk to human handlers (27). Even though our facility excels at handling cervids under research conditions (19, 20), the cumulative risk to animals and people is high when doing frequent manipulations of potential fractious elk for development of a disease model.

Fortunately, a sheep model of bovine DD provides a viable alternative to large animal models (15). In comparison to larger animal models, sheep are easier to handle, are less expensive to maintain, and previous work has demonstrated that *Treponema*-driven bovine DD can be reproduced in a sheep model (15). The primary objective of the current study was to determine if material from TAHD lesions in free-roaming wild elk of the western U.S. would induce similar lesions in a sheep model. In our attempt to fulfill Koch's postulates, we used elk lesion tissue homogenate in the ovine model, characterized primary lesions, and examined lesion material for evidence of *Treponema* species as previously reported in elk TAHD lesions.

## Methods

### Ethical Statement

All animal procedures were approved by National Animal Disease Center Institutional Animal Care and Use Committee in accordance with the standards established by: Public Health Service Policy “US Government Principles for the Utilization and Care of Vertebrate Animals Used in Testing, Research, and Training,” the Guide for the Care and Use of Laboratory Animals (National Research Council), the Animal Welfare Act (1966), and the Guide for the Care and Use of Agricultural Animals in Research and Teaching (USDA, Federation of Animal Science Societies).

### Lesion Material Collection

**Figure 1:**
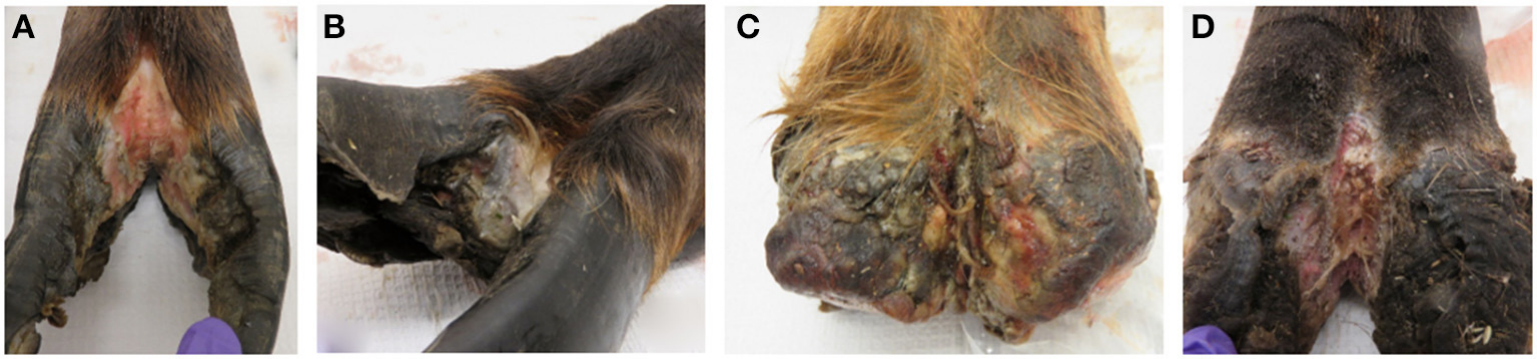
Elk TAHD lesions used to generate lesion homogenate inoculum. **(A)** Lesion grade 3 from hind foot of adult elk cow. **(B)** Lesion grade 2 from front hoof of adult elk cow, same animal as in **(C)**. **(C)** Lesion grade 4 from hind foot of adult elk cow. **(D)** Lesion grade 4 from hind foot of adult elk cow.

Lesion material was collected from four feet (three hind and one front foot) of three adult animals with clinical signs of TAHD within the known endemic area (Hoquiam and Oakville, Washington) by Washington Department of Fish and Wildlife biologists as part of humane removal programs. The four hooves were characterized as lesion grades 3, 2, 4, and 4 in accordance with a previously described scoring system (3) (Figure 1). Hooves were shipped on ice packs overnight to our laboratory in Ames, IA, where lesion tissue homogenates were prepared from active lesion sites as previously described (15, 28). Briefly, debris was removed from hooves using sterile phosphate buffered saline solution (PBS, pH 7.2), and sections of lesions were removed using sterile instruments. After transfer to an anaerobic chamber (Coy Laboratory Products, Grass Lake, MI), tissue was homogenized in Oral Treponeme Enrichment Broth (OTEB, Anaerobe Systems, Morgan Hill, CA). Sterile glycerol and serum (bovine, horse, and rabbit serum mixed in 2:1:1 ratio, Gibco) were added to tissue homogenate and filtered through several layers of sterile gauze. Spirochetes were numerated by direct count method and adjusted to approximately 1 × 10^7^ per ml. Lesion homogenate was frozen at −80°C in 5 ml aliquots for later use. Lesion material from a front and rear hoof of one animal collected in June 2017 was used to prepare material designated as lesion homogenate WA I. Material collected from the hind feet of two different animals in October 2017 was used and the combined lesion homogenates derived from a single hoof from each animal was designated as lesion homogenate WA II.

### Study Design

Fifteen white-faced crossbred sheep (*Ovis aries*) ranging in age from 6 to 12 months were obtained from a local source. Sheep included both castrated males and virgin females. Before initiation of the study, hooves were trimmed and examined for signs of disease or other abnormalities and sheep were evaluated for soundness. Sheep were housed in an outdoor concrete paddock with deep straw bedding under the covered half of the paddock, with free access to water and grass hay. In order to emulate the low nutritional status of elk in the greater Mount Saint Helens area (1), no concentrate or grain was fed during the study. Body condition was monitored by visual inspection or by palpation of the backbone and assessment using a five-point scale (http://smallfarms.oregonstate.edu; accessed 12/28/15). Animals were maintained at body condition scores ≥ 2. Sheep were non-randomly assigned to three treatment groups: those receiving lesion homogenate WA I and those receiving lesion homogenate WA II, and a mock process control group. To ensure variances in body size/ages were equally distributed across treatment groups, sheep were sorted largest to smallest by weight and every third sheep was selected for the mock-inoculated group. All experimental groups (inoculated and mock-inoculated) were housed together for the duration of the study.

### Experimental Overview

The experiment used 15 sheep total, two groups of 5 inoculated/infected sheep elk TAHD lesion-derived material and 5 mock-inoculated, negative controls. Two hind feet were used on each sheep and lesion development was evaluated at 2-, 4-, and 7-weeks post-inoculation. Group sizes and infectivity time (weeks PI) were based on previous data using bovine DD lesion material in which a < 90% infectivity rate was observed, and average time of lesion development was ~4 weeks PI (15).

### Preparation and Wrapping of Inoculation Site

Feet were prepared and wrapped as previously described (15). Three days prior to inoculation, hair and wool on both hind legs were clipped to above the hock. Sheep were restrained and inverted using a sheep-specific tilt-table (Sydell Inc., Burbank, SD), allowing access to feet and legs. Two regions, each approximately 1.5 cm^2^, on each side of the pastern area, were abraded using a rotary tool with tungsten bit. Cotton cast padding, ~40 x 40 x 10 mm, was saturated with a mixture of 50% phosphate buffered saline (PBS) (pH 7.2) and 50% clarified sterile bovine rumen fluid and placed over the abraded area. A piece of small tubing with syringe port (SureFlo^®^ Winged Infusion Set with needle portion removed, Terumo Corporation, Tokyo, Japan) was sandwiched in duct tape and placed so that the tip was over the cotton padding. Cotton and tubing were secured with a layer of brown “cling” gauze (Jorgensen Laboratories, Loveland, CO) followed by plastic film applied to retain moisture and exclude air. Next, a layer of Vetwrap^®^ (3M Animal Care Products, St. Paul, MN) was applied for cushioning followed by an overlapping layer of Gorilla Tape^®^ (Gorilla Glue, Cincinnati, OH). An additional round of Vetwrap^®^ was used to secure and cover the injection port of the tubing. Wraps were checked twice per week for integrity. Gorilla Tape^®^ was reapplied as needed. Every 7 days ± 2 days, 5 ml of 50% Anaerobe Basal Broth (Oxoid, Basinstoke, UK), 25% clarified bovine rumen fluid, and 25% PBS was added via the catheter port to maintain the wet, anaerobic environment in the pastern region. Three days following initial application of wet wraps, inoculation with lesion material or sterile OTEB media was performed once through a small incision made at the bottom of the wrap. One ml of prepared inoculum was placed under the cotton padding via 1 mL slip-tip syringe and the wrap was re-sealed with tape.

### Clinical Observations

Sheep were observed twice daily, and feet were monitored no less than twice each week for development of clinical signs including lameness (altered gait or non-weight bearing posture), evidence of heat or tenderness on palpation, and the presence of atypical odor associated with the bandaged foot. Frequency of lameness observations increased as clinical signs developed. Meloxicam (1-1.25 mg/kg, orally, every 24 h) was given for analgesia once animals demonstrated non-weight bearing lameness in at least one hind limb and was continued as long as lameness was observed or until euthanasia. Personnel assessing clinical signs and administering analgesics were not blinded to the treatment group of the individual animals.

Both feet from all animals were unwrapped at 2 and 4 weeks PI and observed for lesion development. Photographs were taken of feet for documentation of lesion development. Photographs of feet observed at 2, 4, and 7 weeks PI were scored as no lesions, mild, moderate, or severe. Mild lesions were defined as a lesion area that was less than the original scarified area, often with a decrease in erythema and evidence of healing. Moderate lesions were defined as lesions or erosions that were equivalent to the original scarified area and extending into an adjacent pastern area, dewclaw area, coronary band, or heel bulb or hoof sole but not covering > 30% of the total surface area of pastern/dewclaw area, heel bulb, or hoof sole. Severe lesions were defined as being inclusive of the above and encompassing two or more areas and >30% of the total surface area of pastern/dewclaw area, heel bulb, or sole covered in lesion or erosions. Lesion development observations were made on the foot level, with the assumption that 2 feet on the same animal were not independent observations. Wraps were reapplied. Animals were euthanized at 7 weeks PI and hoof samples collected.

### Histology

One strip per foot, ~10 mm wide from the base of the dewclaw, through the center heel bulb to the center sole, was excised, encompassing lesions and adjacent healthy skin, and were preserved in 10% neutral buffered formalin. After fixation, the entire tissue sample (skin and attached keratinized hoof horn) was de-calcified with Kristensen's decalcifying formic acid solution (50% 1 N sodium formate, 50% 8 N formic acid). Decalcification was monitored through chemical testing (29). Briefly, decalcifying solution was tested each day for completion as follows. The decalcifying solution (5 ml) was neutralized with 0.5 N sodium hydroxide, and then 1 ml of 5% ammonium oxalate was added. If the resulting solution became turbid, decalcification was not complete, and tissues were placed in fresh Kristensen's solution. This was repeated each day until the chemical test was no longer turbid, indicating decalcification was complete. Once decalcification was complete, hoof/tissues were soaked in Fibrous Tissue Soaking Solution (100 ml Tween 80 in 1,000 ml 1N hydrochloric acid) (30). Tissues were tested daily for softness (pliability). Depending on the size of the tissues, this process took between 3 and 10 days. At this time, tissues were routinely processed, paraffin embedded, and 5 μm thick sections were stained with hematoxylin and eosin (H&E). Adjacent 5 μm sections were stained using Steiner and Steiner silver stain for visualization of bacteria. Slides were examined by a pathologist blinded to the identity of the study groups. Observations were made for each individual foot, with both feet from the same animal considered to be not independent observations.

### PCR

To test lesions for the presence of treponemes associated with DD and TAHD (3), swabs of lesion sites were collected at 2, 4, and 7 weeks PI. After removal of all bandages, a sterile, dry cotton swab (Puritan Medical Products Company, Guilford, ME) was rubbed over the abrasion or lesion for 15 s. Resulting swabs were placed inside individual sterile polystyrene tubes and transported to the laboratory. The tip of the cotton swab was cut off using sterile scissors and DNA extracted using Qiagen DNeasy Blood and Tissue kit (Qiagen, Germantown, MD) following the manufacturer's instructions for bacteria. Due to the absorption by the cotton, an additional 200 μl of lysing buffer (ATL) and 20 μl of proteinase K were added during the initial lysis step in the first incubation. DNA from swabs from each foot were maintained separately and evaluated on the individual foot level. DNA was quantified using Qubit^TM^ Fluorometric Quantitation (ThermoFisher, Waltham, MA). PCR for *Treponema medium, Treponema pedis, Treponema phagedenis, Fusobacterium necrophorum*, and *Dichelobacter nodosus* was performed in a two-stage nested PCR assay using primers and conditions as previously described (15) and presented in Supplementary Table S1. Positive controls were genomic DNA from each of the respective bacteria, details of strains used are given in Supplementary Table S1, and negative control was PCR reaction mixture with sterile water. PCR reaction products were visualized by electrophoresis on 1.5% agarose gel stained with SYBR Safe DNA gel stain (Invitrogen Life Technologies, Carlsbad, CA).

### Bacterial Observation

Microscopic observations: At 2, 4, or 7 weeks PI, sterile cotton swabs of the lesion sites as described above were placed in sterile 5-ml polystyrene tubes and transported to the laboratory. Swabs from each foot were maintained separately and evaluated on the individual foot level. Approximately 0.5 ml sterile PBS was added to each tube and incubated at room temperature with periodic vortex for 1 h, then 10 μl was placed on a clean glass slide and viewed using dark-field microscopy to examine for the presence of spirochete-shaped bacteria.

### Enzyme-Linked Immunosorbent Assay

Whole blood (8-10 ml) was collected *via* jugular venipuncture into serum separator vacutainer tubes (BD Vacutainer SST, Franklin Lakes, NJ) prior to inoculation (week 0) and 6 weeks PI. Blood was allowed to clot, and serum was separated by centrifugation (Beckman Coulter Avanti J-E with JS-5.3 rotor, 700 × G, 20 min, 4°C), then stored at −20°C. *T. phagedenis, T. medium*, and *T. pedis* were grown in OTEB to confluence, bacterial cells harvested and washed by centrifugation, and antigen prepared from whole-cell sonicates as described previously (15). Mixed treponeme antigen (6 μg whole cell dry weight/ml in PBS) was bound overnight to a 96-well plate (Corning^®^ 96-well Clear Flat Bottom Polystyrene High Bind Microplate #9018). Plates were blocked with 45 μg/ml porcine IgG (Sigma-Aldrich, St. Louis, MO) in PBS with 0.05% (v/v) Tween 20 (Sigma-Aldrich, St. Louis, MO) (PBST) overnight. Serum was serially diluted, added to plates, and incubated for 1 h at 37°C then at 4°C overnight. Plates were washed three times with PBST. Bound antibody was detected by horseradish peroxidase-conjugated rabbit anti-sheep IgG (γ-chain) (KPL, Gaithersburg, MD), incubated 1 h at 37°C. Substrate was SureBlue Reserve TMB Microwell Peroxidase Substrate (KPL, Gaithersburg, MD) and reaction stopped with TMB stop solution (R&D systems, Minneapolis, MN). Plates were read at 455 nm using SpectraMax M2 plate reader (Molecular Devices, San Jose, CA). Titer was reported as the lowest dilution with an optical density equal or greater than the mean plus two standard deviations of wells containing only PBS. Assay was performed in duplicate by a technician blinded to treatment groups and lesion outcomes for individual animals. ELISA results were analyzed using Prism 6 (GraphPad Software, San Diego, CA) statistical software fitting a two-way ANOVA for repeated measures to log transformed data with and Tukey's multiple comparisons for simple effects within columns (between inoculum groups) and between rows (within inoculum groups, between timepoints).

### 16S rRNA Gene Sequencing and Data Analysis

Using DNA extracted from lesion swabs collected prior to infection (week 0), and at 2, 4, and 7 weeks PI, amplicon library preparation for sequencing of the bacterial 16S rRNA gene v4 region was completed according to standard methodology at Argonne National Laboratory (https://www.anl.gov/bio/environmental-sample-preparation-and-sequencing-facility). Amplicons were sequenced on an Illumina HiSeq 2500. Universal 16S rRNA gene forward primer (515F) and unique Golay barcoded reverse primers (806R) were used, and amplification was as previously described (31).

Reads for each individual sample were demultiplexed and binned according to barcode by idemp (https://github.com/yhwu/idemp). Reads were checked for quality and processed into operational taxonomic units (OTUs) using mothur analysis software (v.1.43.0) following the strategy outlined by Kozich et al. and https://mothur.org/wiki/miseq_sop/ (accessed January 2021) (32, 33). The 16S rRNA gene v4 sequences were aligned to the Silva bacteria reference database (release 132) and taxonomy was assigned based on the mothur-formatted version of the RDPtraining set (trainset16_022016). Sequences were clustered into OTUs based on the pairwise distances between the unique sequences. Further data analyses and statistical comparisons were made using R and the following R-packages “DESeq2,” “metacoder,” “microbial,” “OTUbase,” “phyloseq,” “taxa,” and “vegan.” The analyses also employed the following general R-packages: “dplyr,” “ggplot2,” “gplots,” “magrittr,” “RColorBrewer,” “reshape,” “scales,” “tidyverse,” and “VennDiagram” (https://CRAN.R-project.org/package=microbial) (https://CRAN.R-project.org/package=vegan) (32, 34–37). The data were rarefied before comparative analyses except for the DESeq2 analysis that was performed with the unrarefied data.

### Data Management

Raw data collected from histology report, ELISA, PCR, gross and microscopic observation, and representative photographs of each foot at each timepoint accompany this manuscript in the Supplementary Data Files. 16S rRNA gene sequencing data (fastq files) can be accessed under NCBI Bioproject accession number PRJNA781217 (https://www.ncbi.nlm.nih.gov/bioproject).

## Results

### Clinical Presentation and Histology

Five sheep were inoculated with elk hoof lesion batch 1 (WA I) and five sheep were inoculated with elk hoof lesion batch 2 (WA II). Two animals in WA I group were removed from the study, one due to injury unrelated to the experiment at 3 weeks PI and one due to penetration of severe lesions into tendon and muscle structures beneath the dermis occurring at 5 weeks PI. There were no observed differences in lesion development between WA I and WA II inoculums. For this reason, data from both inoculums were combined and data are presented as elk inoculum (*n* = 10 sheep with 8 surviving to 7 weeks, 16 feet) or mock inoculated (*n* = 5 sheep, 10 feet) groups.

**Figure 2:**
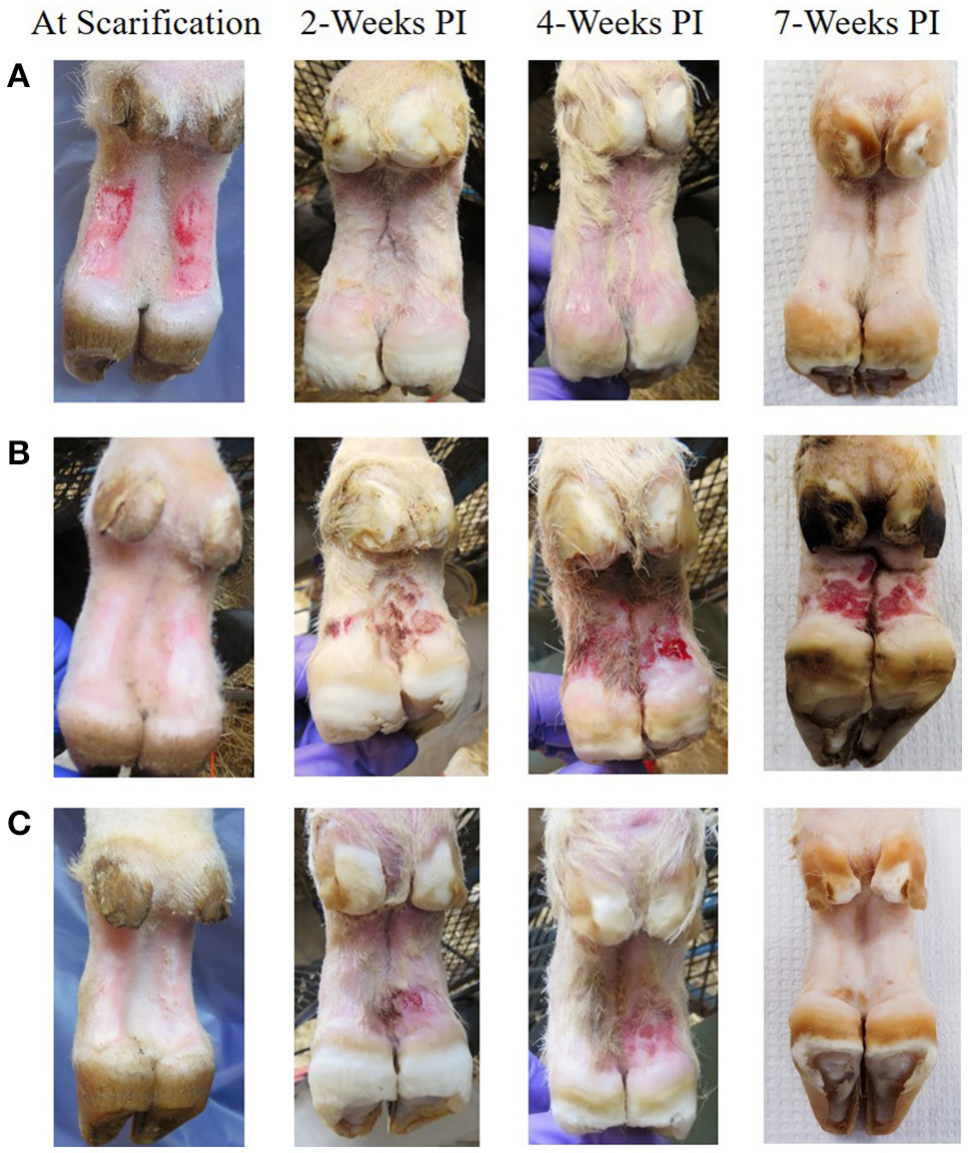
Representative photographs of mock inoculated and inoculated feet showing lesion progression from scarification to 7 weeks PI. **(A)** Mock inoculated, **(B)** Elk TAHD lesion inoculated which maintained lesions through 7 weeks PI, **(C)** elk TAHD lesion inoculated spontaneously resolved before 7 weeks PI.

**Figure 3:**
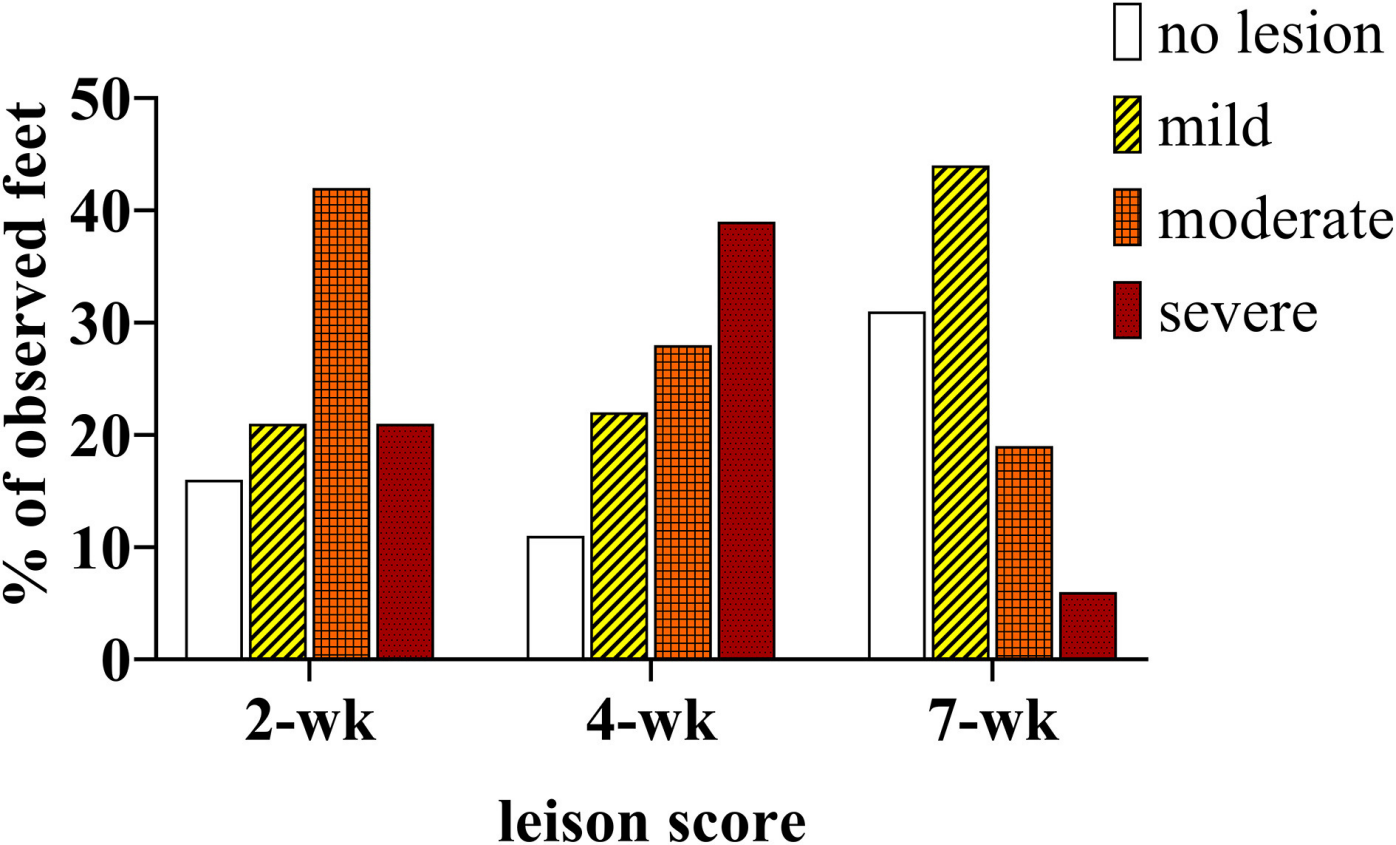
Percentage of observed feet in each lesion severity category at 2, 4, and 7 weekS PI. All mock inoculated feet were non-lesioned or healthy looking at all timepoints (not shown), TAHD inoculated feet were scored as lesions (white bars/open bars), mild lesions (yellow bars/diagonal lines), moderate (orange bars/cross-hatched), or severe lesions (red bars/dotted).

At 2 weeks PI all mock inoculated control animals had healed from the abrasion procedure and no lesions or spirochetes were observed for the remainder of the trial (Figures 2, 3; Table 1). Representative photographs of lesion progression are given in Figure 2. Photographs of all feet are provided in Supplementary Figure S2. Photographs of all feet were scored categorically and proportions of each category per inoculum at each timepoint are presented in Figure 3. At 2 weeks PI, almost all (85%) of the infected animals had developed lesions, with most classified as either moderate or severe (42 and 21%, respectively) (Figure 3). The lesion severity increased at 4 weeks PI with 29% observed feet with moderate lesions and 29% with severe lesions. However, this trend reversed at week 7 with many feet demonstrating resolution of lesions. At this time, 31% of observed feet had no lesions and 44% were scored as having mild or resolving lesions. In general, observation of spirochetes in swab fluid correlated with the presence of lesions (Table 1, Supplementary Data File).

**Table 1:** Number of observed feet positive for spirochetes in swab fluid.

**Number of feet positive/number** of feet observed^*^	**2 week PI**	**4 week PI**	**7 week PI**
Mock	0/10	0/10	0/10
Inoculated	9/18	16/18	11/16

* *Minimum of 10 microscope fields observed*.

Histopathological examination of the wrapped feet identified changes in the skin environment caused by moist wraps were exhibited by samples from mock infected controls. Lesions associated with TAHC lesion material included mild erosions, mild hyperkeratosis, mild to moderate proliferative acanthosis, mild to moderate superficial lymphoplasmacytic inflammation, moderate dermal edema and alopecia, and numerous superficial coccobacilli and plump rods extending into dermal erosions. The full histopathology report is in the Supplementary Data File.

### ELISA

**Figure 4:**
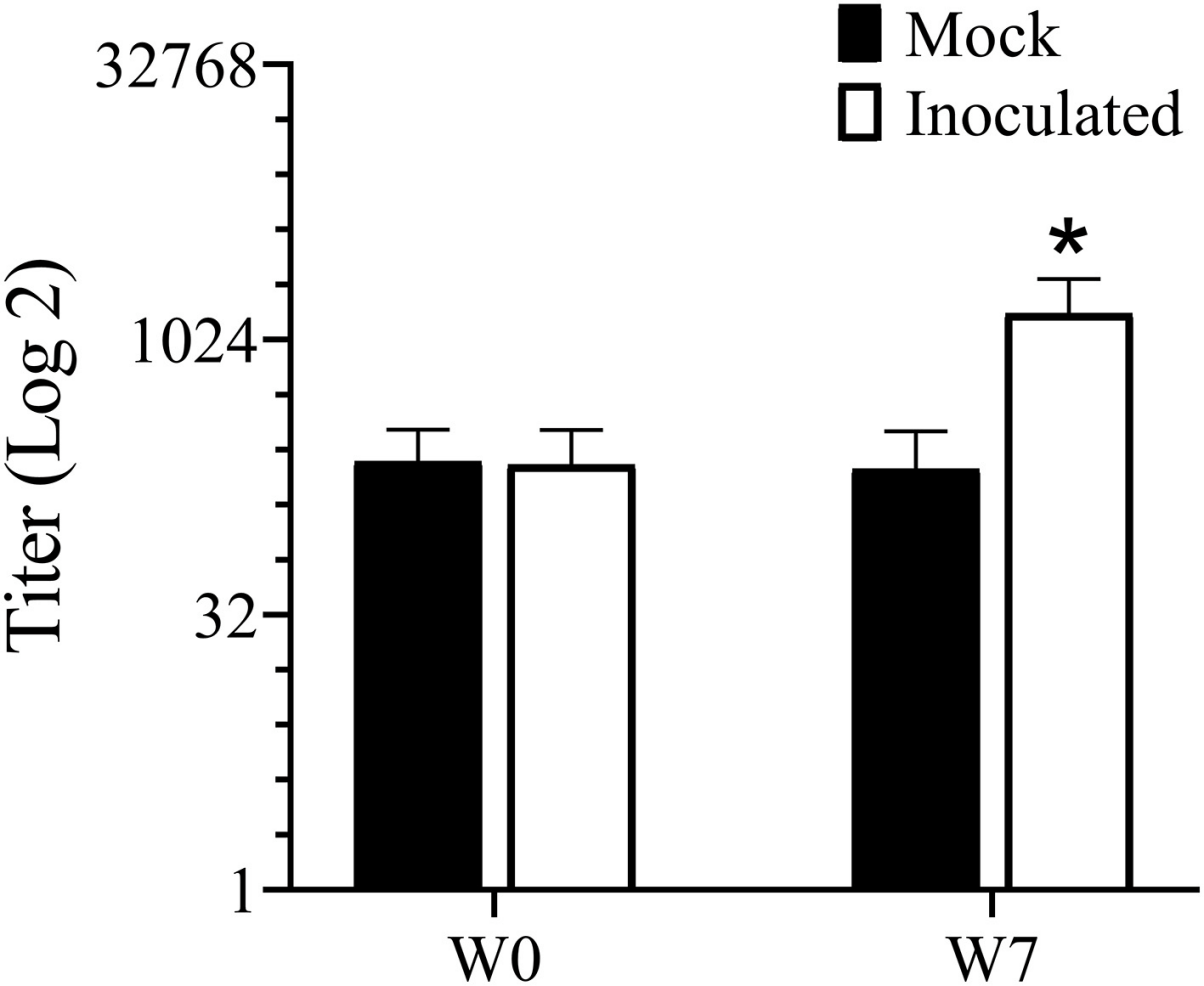
ELISA for serum antibody against mixed *Treponema* antigen measured prior to and 7 weeks PI. *Indicates statistical significance from both week 0 as well as mock inoculated group (*P* < 0.05).

Antibodies to *Treponema* are usually an indication of exposure and titers tend to correlate well with lesion severity (38). Serum antibody titers increased (*P* < 0.05) in the inoculated group but not in the mock infected group between week 0 (pre-infection) and week 7 (Figure 4).

### PCR

*T. medium, T. pedis*, and *T. phagedenis* have consistently been found within TAHD lesions (2, 3) as well as within bovine DD lesions (2, 3, 15, 31, 39–47). Using species specific primers, one or more of these species were identified on inoculated foot swabs in at least one sampling timepoint (Table 2). All foot swabs were negative for *Treponema* species prior to inoculation (week 0). Most feet were positive for *Fusobacterium* at multiple timepoints, including samples from mock inoculated and from samples obtained prior to inoculation with lesion material (week 0). While *Fusobacterium* is a known contributor to foot rot of cattle and small ruminants, its role in DD is not well defined and is controversial (11, 41, 48–54). Our data supports an ambiguous role for *Fusobacterium* in TAHD as most sheep feet, even in the mock inoculated treatment group, were positive for *Fusobacterium* prior to inoculation and many in the control group did not develop lesions. Animals were sorted into treatment/infection groups prior to *Fusobacterium* presence on feet was known. Only two samples, from the same inoculated animal at 7 weeks PI, was positive for *Dichelobacter*, indicating that it was not a key factor in this disease. Several mock inoculated feet were PCR positive for *T. medium* and *T. phagedenis*, most commonly at 7 weeks PI. No lesions were observed on mock-inoculated feet at week 7, nor spirochetes in silver-stained histopathology slides. These data should be interpreted carefully considering the observation that PCR techniques used in the current study are sensitive to cross-contamination which may have been influenced by housing all sheep in the same pen. Research staff does handle animals in a manner to reduce any cross-contamination (i.e., working non-infected controls first or on separate days, disinfection of gloves and equipment between groups); however, sometimes excessive rain or adverse conditions work against us (55, 56).

**Table 2:** Number of PCR positive reactions over the number of sampled lesion swabs prior to scarification (0 week) and at 2, 4, and 7 weeks PI.

	***T. pedis***			
**0 week**	**2 week**	**4 week**	**7 week**	
Mock	0/10	1/10	1/10	0/10
Inoculated	0/20	9/20	16/18	4/16
***T. medium***				
**0 week**	**2 week**	**4 week**	**7 week**	
Mock	0/10	0/10	3/10	0/10
Inoculated	0/20	1/20	15/18	9/16
***T. phagedenis***				
**0 week**	**2 week**	**4 week**	**7 week**	
Mock	0/10	0/10	4/10	1/10
Inoculated	0/10	14/20	15/18	16/16
***Fusobacter***				
**0 week**	**2 week**	**4 week**	**7 week**	
Mock	8/10	0/10	4/10	4/10
Inoculated	15/20	18/20	14/18	16/16
***Dichelobacter***				
**0 week**	**2 week**	**4 week**	**7 week**	
Mock	0/10	0/10	0/10	0/10
Inoculated	0/20	0/20	0/15	2/14

### 16S rRNA Gene Sequencing

For 16S rRNA gene analysis, 119 samples representing four timepoints, two inoculum types (either elk lesion homogenate or mock), and 15 sheep (both right and left feet) were sequenced with 54,615 average number of read pairs per sample (16,137 min, 75,369 max). Prior to comparative analyses, the data were subsampled to 14,113 sequences, which was the smallest number of processed 16S rRNA gene v4 sequences among the samples. The DESeq2 analysis used the full (i.e., not subsampled) dataset.

**Figure 5:**
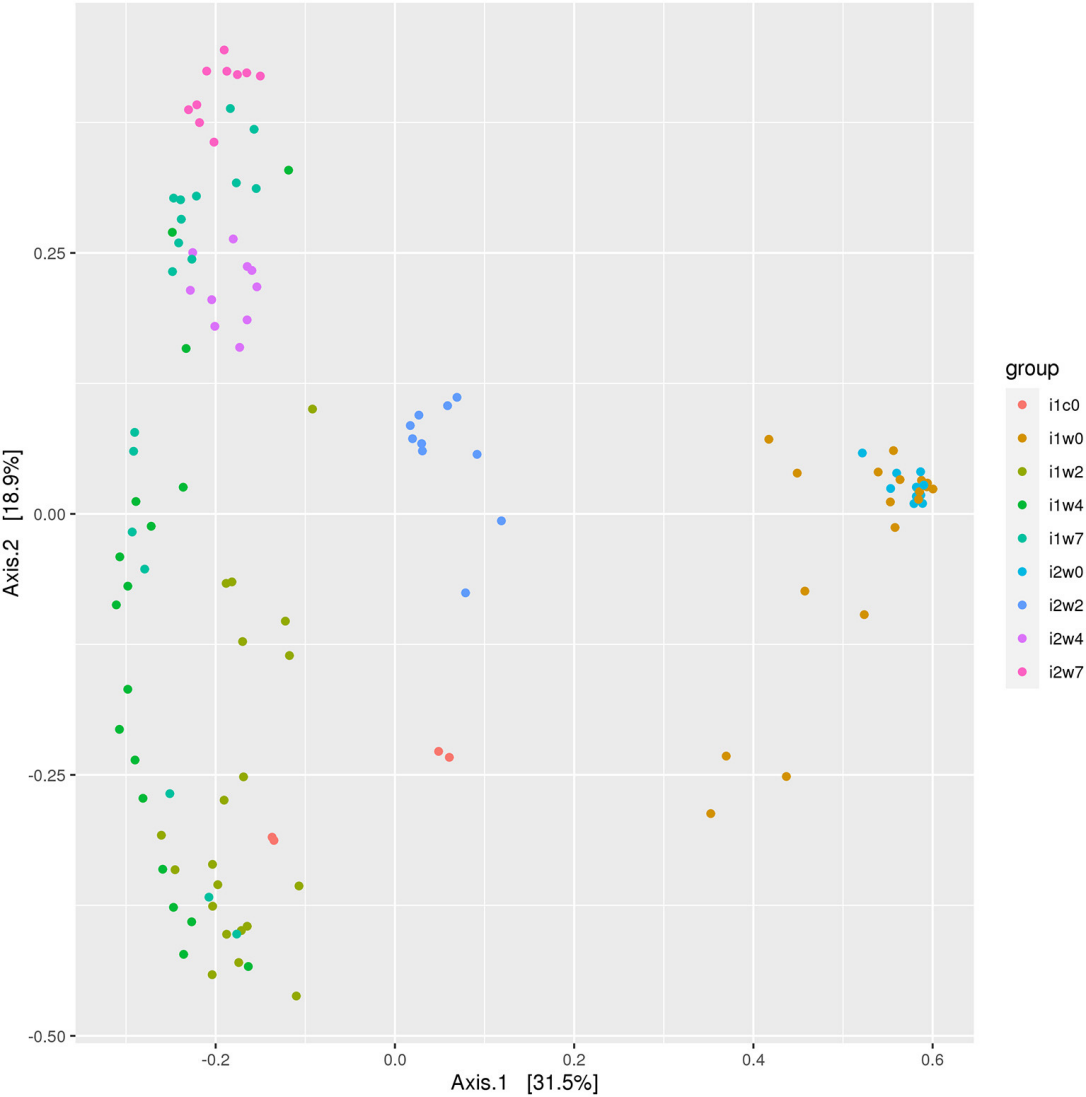
Beta diversity ordination plot. Bray-Curtis Principal Component Analysis of bacterial 16S rRNA gene sequencing OTU grouped by inoculum and weeks. Elk lesion homogenate (i1c0), lesion inoculated sheep feet at 0 weeks (i1w0), 2 weeks (i1w2), 4 weeks (i1w4), and 7 weeks (i1w7) post-inoculation and mock inoculated 0 weesk (i2w0), 2 weeks (i2w2), 4 weeks (i2w4), and 7 weeks (i2w7) post-mock inoculation.

Data were analyzed by inoculum type: elk lesion material, infected, or mock inoculated, and by timepoints (0, 2, 4, and 7 weeks PI). Alpha diversity was most similar at week 0 when, theoretically, all sheep feet were the same. High similarity was also observed between inoculum and inoculated feet at week 2 (Supplementary Figure S3). Beta diversity analysis indicates overlap of clustering of samples at week 0, but divergence at week 2 between elk lesion inoculated and mock inoculated samples (Figure 5). Further divergence of the bacterial community was noted at week 4 as TAHD-inoculated samples more closely resembled the original elk lesion material and clustered less with week 0 or mock-inoculated sheep samples.

**Figure 6:**
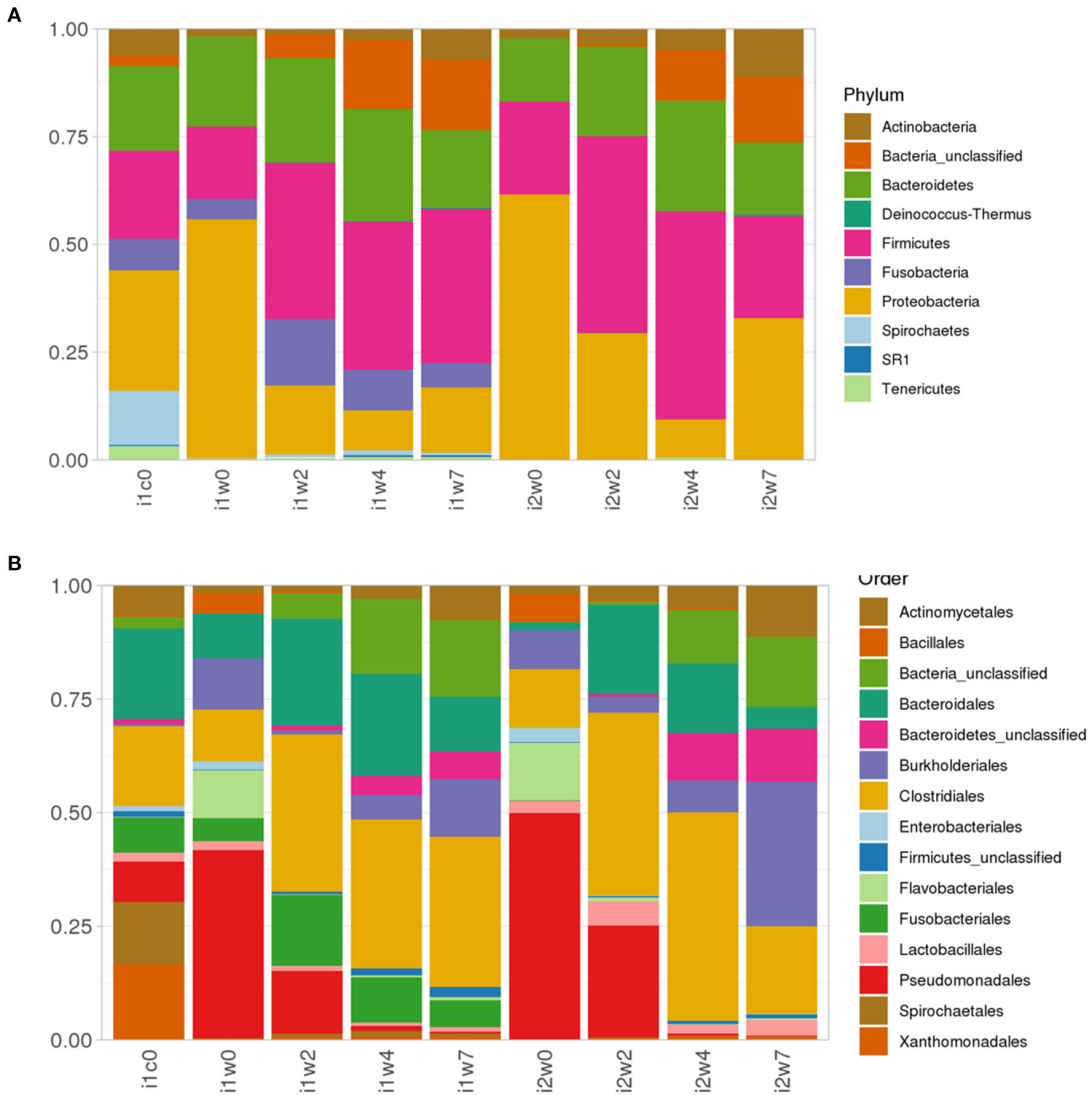
Relative abundance of **(A)** top 10 Phyla taxonomic bacterial groups and **(B)** top 15 order in elk lesion homogenate (i1c0) and swabs from inoculated (i1) and mock-inoculated sheep (i2) feet at 0, 2, 4, and 7 weeks post-inoculation.

Most abundant phyla in each inoculum type and timepoint are depicted in Figure 6. Week 0 samples are dominated by phyla Proteobacteria, order Pseudomonadales, and phlya Firmicutes, order Clostridales, with a strong component of Bacteroidetes (Bacteroidales) represented across all samples. Spirochetes and Spirochaetales were a dominant phylum in the elk lesion homogenate, and they remain in the top 10 Phyla of the inoculated samples at post-inoculation timepoints (2, 4, and 7 weeks PI) (Figure 6).

**Figure 7:**
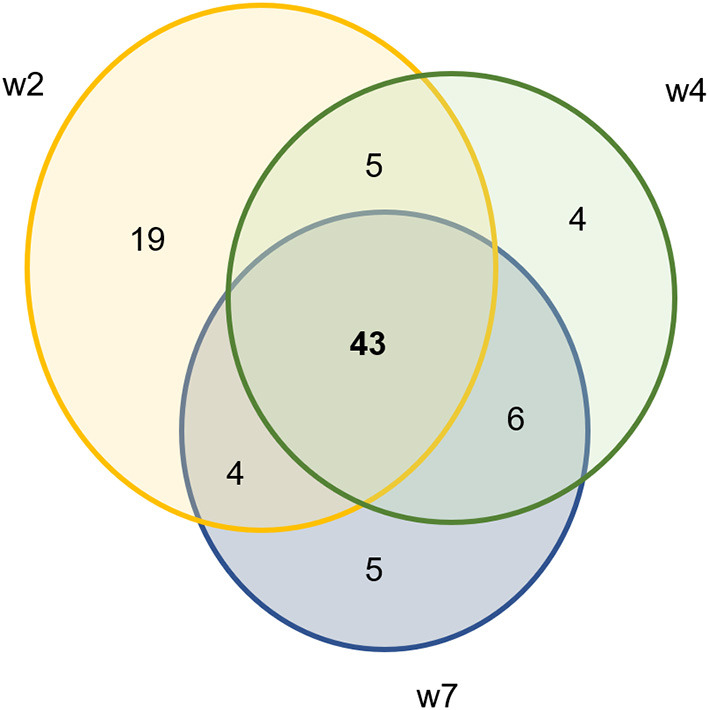
Ven diagram of OTUs that are significantly positively overrepresented in infected samples with mean count > 20.

**Figure 8:**
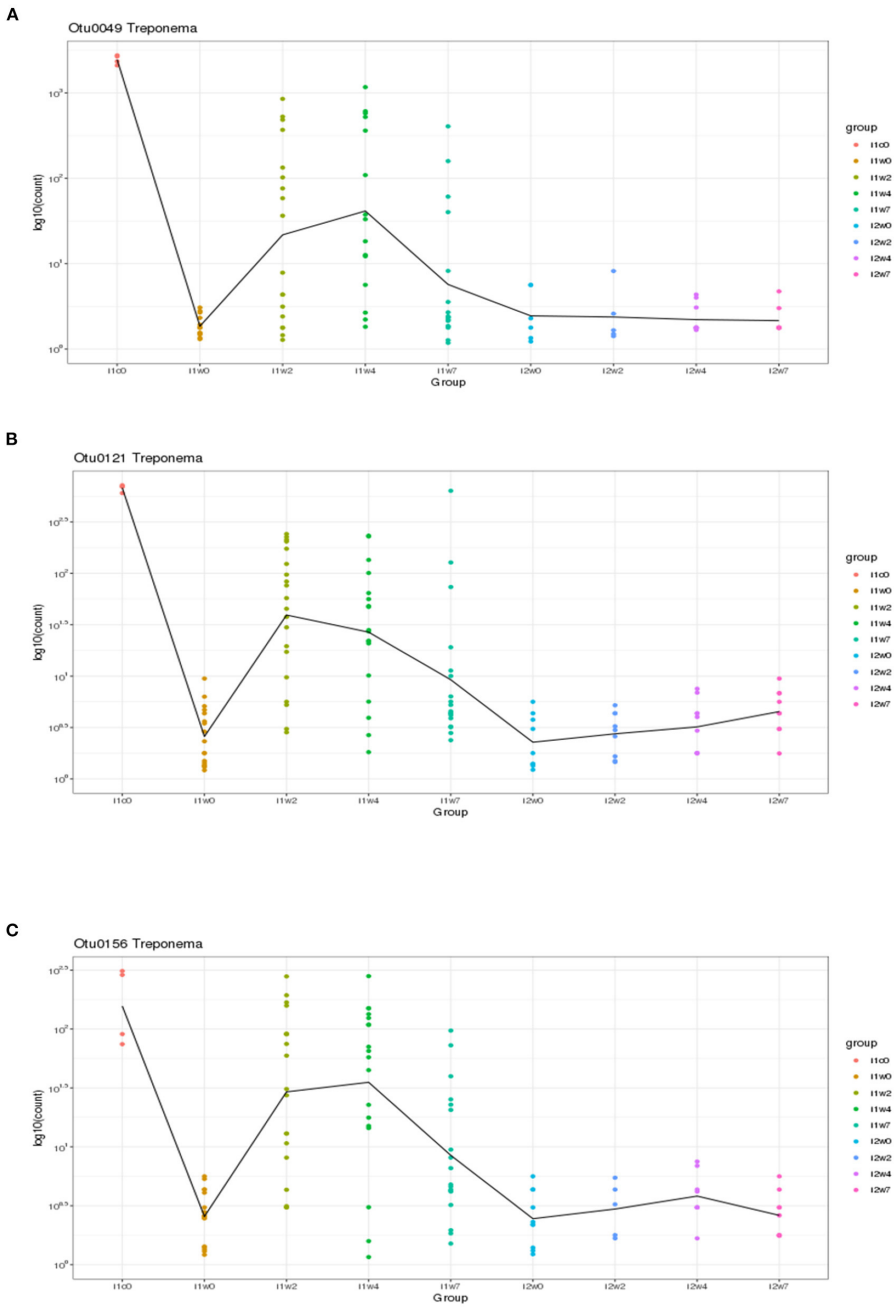
Three *Treponema* genus OTUs which are significantly more abundant in two or more timepoints (weeks 2, 4, and/or 7) in inoculated feet as compared to mock feet. **(A)** Otu0049, **(B)** Otu0121, and **(C)** Otu0156.

Using DESeq2, pairwise analysis was made of timepoints between the inoculated and mock samples. There were 86 OTUs found to be positively significantly overrepresented in inoculated samples at 2, 4, or 7 weeks PI and have a base mean count > 20. Figure 7 shows the distribution of these samples across the timepoints. There were 43 OTUs present at all three timepoints meeting these criteria (significantly different between mock and inoculated, increased in inoculated with base mean count > 20) including 3 *Treponema*, 1 Actinobacteria (*Actinomyces*), 4 unclassified Bacteria, 10 Bacteroidetes (including 4 *Porphyromonas* and 1 *Prevotella*), 21 Firmicutes (Clostridia, Peptostreptococcus, Filifactor, and Ruminococcaceae), 1 Fusobacteria, 1 Proteobacteria (*Campylobacter*), SR1, and 1 Tenericutes (*Mycolpasma*). As represented by the treponemes (Figure 8) OTUs Otu0049, Otu0121, and Otu0156, the OTUs are present in the elk lesion material and inoculated samples as lesions develop (2, 4, and 7 weeks PI) but not with samples obtained prior to inoculation (week 0) or mock samples at 2, 4, or 7 weeks. Plots showing the representation of individual OTUs by sample and relative abundance for the other 40 OTUs can be found in Supplementary Figures S4-S6.

## Discussion

The goal of this experiment was to evaluate the ability of TAHD lesion material from elk to induce disease in a sheep model.

Using lesion material collected from wild elk within the endemic disease area exhibiting classic TAHD lesions, we were able to produce lesions in most sheep by week 4 PI. Lesions contained spirochete shaped bacteria and molecular analysis determined that *Treponema* species or phylotypes typically associated with TAHD and livestock DD were associated with lesions. Spirochete shaped bacteria were at the leading edge of the lesions and accompanied by neutrophilic inflammatory infiltrates, and keratinocyte erosion and proliferation in accordance with typical histological presentation of TAHD (3). However, gross appearance of lesions in the sheep model differed from natural infection in elk, as lesions originated in the interdigital area on the front of the foot and then extended into or under the hoof horn wall. Inoculation of a sheep model with TAHD material resulted in a lesion pattern similar to that produced by bovine DD material, with lesions originating at the scarification sites in the pastern area. In this study, sheep appeared to be naïve to the treponeme spirochetes, as serum antibody titers increased in THAD infected sheep but not in mock inoculated controls. It should be emphasized that the sheep model is an acute model for studying induction and early pathogenesis of lesion development but is probably not adequate for characterizing chronic stages of disease when more extreme gross lesions such as hoof capsule sloughing are observed. However, the containment provided by the extensive wraps in the sheep model allows study of early lesion development including evaluation of invading bacteria without confounding effects of soil or manure contamination.

In addition to the presence of *Treponema*, sequencing of bacterial 16S rRNA genes from lesions produced in the sheep model demonstrated the consistent presence of other bacterial phylotypes associated with TAHD and DD. These included bacteria in *Porphyromonas, Prevotella*, Bacterioidetes, *Actinomyces, Campylobacter*, and *Mycoplasma* genuses. Comparing mock to inoculated tissues at three timepoints, 86 OTUs were increased in inoculated samples with the majority found in all three sampling (43 OTUs) timepoints but the highest number was found at week 2 (Figure 7). Others have reported that microbial diversity of DD lesions decreases with time, with treponemes eventually becoming the dominant organism (31, 41, 44, 57). This was not observed in the current study. TAHD may differ from DD in this. Future studies of TAHD are needed to characterize microbial diversity or dominance with different lesion grades or time after infection with TAHD lesion material. Previous studies have identified bacteria in the complex community of DD lesions and have suggested roles for different bacteria in lesion development. Actinomyces are known for anaerobic infection of compromised skin, forming branched, fusiform, fungal-like mats. Actinomyces have been observed in TAHD tissues and may be mistaken for spirochetes on observation (1, 3) (Supplementary Figure S4B). *Fusobacteria* are a causative agent of foot rot in ungulates and have been strongly associated with DD. In our experiment, we detected Fusobacterium in nearly all inoculated feet and just less than half of the mock inoculated samples prior to inoculation (Table 2). Otu0002, identified as a *Fusobacterium* species, was significantly over-represented in TAHD inoculated feet (Supplementary Figure S4F), indicating a probable positive correlation with lesion development. In foot rot of livestock, Fusobacterium is found in conjunction with other bacteria, most commonly *Dichelobacter nodosus* (58). *D. nodosus* can be transmitted between species (sheep and cattle), especially when common grazing areas are used (59). However, we did not find *D. nodosus* on the wrapped feet using bacterial 16S rRNA gene sequencing analysis or targeted PCR (Table 2).

Proteobacter quantity and diversity decreased the longer the feet were wrapped, regardless of mock or TAHD inoculation. As others have identified a non-typable *Campylobacter* associated with bovine DD lesions (31, 60–62), the observation in the current study that a *Campylobacter* isolate, Out0128, is overrepresented in inoculated feet and increased with time was of interest (Supplementary Figure S4G). SR1 is an interesting taxon, found in sequencing projects from environmental samples to human microbiome (http://bytesizebio.net/2013/03/29/the-power-of-single-cell-genomics-the-mysterious-sr1-bacteria-have-a-unique-genetic-code/). Associated with oral cavity and periodontal disease, it is not surprising to find it associated with TAHD (Supplementary Figure S4H), as there is an interesting overlap of bacterial phylotypes between periodontal disease and TAHD or DD of ruminants.

Clostridia are a very large, varied class and are known for wide distribution across environments, including soil, and human and animal intestinal tracts, with many species being opportunistic pathogens. In the current study, 18 OTUs belonging to the Clostridiales cluster including unclassified Clostridiales, *Filifactor, Peptostreptococcus, Helcoccus, Lachnospiraceae*, and *Clostridiales* Incertae Sendis XI increased in TAHD inoculated samples as compared to mock samples (Supplementary Figure S5). Bacterioidetes are the largest phyla found in the rumen of sheep and cattle. Bacterioidetes phyla includes the genera *Porphyromonas, Prevotella*, and *Odoribacter*. Otus identified as these genera were increased in the TAHD infected samples (Supplementary Figure S6). These bacteria and closely related species have been identified in periodontal disease of humans and dogs, soft tissue abscesses, foot rot, and DD (63). However, specific roles for these bacteria have not yet been identified in TAHD or DD lesion development (63). The role of many of these bacteria in lesion development or continuance is unclear as a few phylotypes identified as *Porphyromonas* increase under a wet foot environment alone (Figure 6, i2w7).

Although spirochete bacteria are usually dominant in DD and can be observed microscopically (Table 1) or identified by targeted PCR (Table 2), 16S rRNA gene sequencing suggests they are a minor portion of TAHD lesions. In the current study we observed that three *Treponema* OTUs, Otu0049, Otu0121, and Otu0156, were overrepresented in infected feet samples after inoculation. This may be influenced by differences in when swabs were collected and the use of swabs for sampling rather than biopsy samples. It should be noted that the size of sheep's feet makes taking repeat biopsies inadvisable (11) and that treponemes are often invasive, found deep within the lesions, and often located at the leading edge of the lesion. Our results and data from others may suggest that, when compared to other bacterial populations, treponemes are not present in large numbers in lesions or readily identified by surface swabs (11). While individual species resolution using only the 16S rRNA gene V4 region has reservations, BLAST search of the three significant *Treponema* OTUs and the three non-significant OTUs (Otu0216, Otu0267, and Otu0276) all gave 98% or greater homology to previously recognized bovine DD *Treponema* species, Otu0049 and Otu0216 with 99 and 97% homology to *T. pedis* (NCBI GenBank ID: KR025849.1), Otu121 with 99 and 98% homology with *T. medium* and *T. vincentii* (NCBI GenBank ID: KP750179.1; KT192153.1; which cannot be resolved using only V4), Otu0156 with 99% homology to *T. phagedenis* (NCBI GenBank ID: CP054692.1), and Otu267 and Otu276 having 98% homology to *T. putidum* and *Treponema* sp. PT8 (which again has 97% homology to *T. pedis*). Therefore, it is very possible as was suggested by the close genetic relatedness of the elk, sheep, and bovine isolates as studied by Clegg et al. that DD regardless of host (bovine, sheep, or elk) the disease is being driven by the same organisms (4). Much like *Fusobacterium* and *D. nodosus*, hoof-associated *Treponema* does not appear to be host species restricted, in fact, the presence of cattle may be a risk factor on sheep farms for CODD (64). Recent publications showing that Mediterranean Buffalo (housed in milking herds) and European Bison (in zoological parks) are also susceptible to DD (65–67) further suggesting that any ungulate species is susceptible given the proper environmental conditions and the presence of these pathogenic bacteria.

Characterizing the role of various bacteria and associated phylotypes was not a primary goal of this study. We did find that bacterial 16S rRNA gene sequencing was a useful method for contrasting microbial communities between mock and inoculated samples. By evaluating the bacterial community both as a whole and using individual OTU contribution, our data suggest a core transmissible bacterial element to TAHD. We cannot explain why TAHD lesions did not persist in the ovine model. Despite attempting to mimic environmental stressors placed on elk under field conditions such as a lower nutritional plane, our results suggest that not all elements of the elk-pathogen relationship were replicated in the ovine model. Although elk hoof samples were shipped and processed quickly, the possibility cannot be eliminated that a key member of the bacterial consortium necessary for chronicity of the lesions was eliminated. Additionally, we recognize the limitations of the sheep model. We utilized a method and inoculation steps that had previously resulted in success, putting the emphasis on testing the transmission and “pathogenesis” of the TAHD bacteria. Alternate inoculation sites (the dorsal aspect of the interdigital space in contrast to the pastern) might have eventually led to gross or clinical lesions similar to those seen in the elk. However, for welfare reasons we were not going to develop the long-term chronic lesions that resulted in the gross pathology associated with TAHD, namely the extreme overly grown, cavitated, and eventual sloughed hooves. Further work characterizing the bacterial populations in TAHD lesions, further development of animal models, and/or continuation of efforts to characterize the epidemiologic characteristics of the disease will be required to develop intervention strategies for reducing disease prevalence in elk in the endemic area.

In this study we were able to experimentally induce lesions with hallmarks of TAHD in a sheep model using lesion material derived from wild elk with TAHD. Histopathology was analyzed by a pathologist who had done the original description of the elk disease. Sheep lesions had strong histopathologic similarity to TAHD in elk, suggesting the same cellular process despite varying in location on the foot. The molecular assays revealed that the bacterial population was transmitted. While imperfect, the model allowed for initiation of early-stage lesions, and demonstrated the transmissible state of the bacterial consortium in a relevant, readily available animal model that has a lower risk of injury to both animals and human workers. Further study on the bacterial community and how individual bacterial members contribute to lesion development is warranted.

## Data Availability Statement

The datasets presented in this study can be found in online repositories. The sequence data files are available at https://www.ncbi.nlm.nih.gov/ under Bioproject TAHD lesion material in sheep model Accession: PRJNA781217 ID: 781217.

## Ethics Statement

The animal study was reviewed and approved by National Animal Disease Center Institutional Animal Care and Use Committee.

## Author Contributions

JW-W, KM, SH, and DA contributed to conception and design of the study. KM provided the elk lesion material. JW-W, DA, and SO performed the study and collected the data. SH performed the pathology analysis. DB performed the 16S rRNA gene sequencing analysis. JW-W wrote the first draft of the manuscript. SH, DB, SO, and DA provided sections of the manuscript, editing, and critical review. All authors contributed to manuscript revision, read, and approved the submitted version.

## Funding

This work was conducted without grants or funds from public or private entities. Work was completed by US Department of Agriculture employees in the course of their assigned duties in relation to project number 5030-32000-223-00-D. Mention of trade names or commercial products in this study is solely for providing specific information and does not imply recommendation or endorsement by the U.S. Department of Agriculture.

## Conflict of Interest

The authors declare that the research was conducted in the absence of any commercial or financial relationships that could be construed as a potential conflict of interest.

## Publisher's Note

All claims expressed in this article are solely those of the authors and do not necessarily represent those of their affiliated organizations, or those of the publisher, the editors and the reviewers. Any product that may be evaluated in this article, or claim that may be made by its manufacturer, is not guaranteed or endorsed by the publisher.
