# Supplementary material for: Lesion Material From Treponema-Associated Hoof Disease of Wild Elk Induces Disease Pathology in the Sheep Digital Dermatitis Model
Source: Front Vet Sci. 2022 Jan 12;8:782149. doi: 10.3389/fvets.2021.782149 (PMC8790030; doi:10.3389/fvets.2021.782149)
Supplement: Supplementary file 2 [file Data_Sheet_2.docx]

| Supplementary data  Sequence data (fasta files) can be located at <https://www.ncbi.nlm.nih.gov/sra?linkname=bioproject_sra_all&from_uid=781217>  TAHD lesion material in sheep model Accession: PRJNA781217 ID: 781217 | | | | | |
| --- | --- | --- | --- | --- | --- |
| Table S1: Nested PCR primers, cycle conditions amplicon product size and positive control strains used in this study. | | | | | |
| Primer Target | Primer Sequence | | Cycle Conditions | Amplicon Size (bp) | DNA from Strain used |
|  | Forward Sequence 5’ to 3’ | Reverse Sequence 5’ to 3’ |  |  |  |
| Bacterial 16S rDNA subunit (Universal) | GAA TGC TCA TCT GAT GAC GGT AAT CGA CG | TAC CTT GTT ACG ACT T | 95°5´, (94°1´, 55°3´, 72°3´) x 25 cycles, 72°7´ | 1526 |  |
| *Treponema phagedenis* | GAA ATA CTC AAG CTT AAC TTG AGA ATT GC | CTA CGC TAC CAT ATC TCT ATA ATA TTG C | 95°5´, (95°1´, 64°1´, 72°2´) x 40 cycles, 72°10´ | 400 | *Treponema phagedenis* strain 4A  (Wilson-Welder, et al., BMC Microbiol  . 2013 Dec 5;13:280. doi: 10.1186/1471-2180-13-280.) |
| *Treponema medium* | GAA TGC TCA TCT GAT GAC GGT AAT CGA CG | CCG GCC TTA TCT CTA AGA CCT TCT ACT AG | 95°5´, (95°1´, 68°2´, 72°2´) x 40 cycles, 72°10´ | 475 | *Treponema medium* G7201 Umemoto et al. (ATCC 700293) |
| *Treponema denticola* | TAA TAC CGA ATG TGC TCA TTT ACA T | TCA AAG AAG CAT TCC CTC TTC TTC TTA | 97°1´, (97°0.75´, 60°0.75´, 72°1´) x 40 cycles, 72°4´ | 316 | *Treponema denticola* a[CIP 103919, DSM 14222] *(ex* Flugge) Chan et al. (ATCC 35405) |
| *Treponema vincentii* | AGA GTT TGA TCC TGG CTC AG | AAT ACT TCT TAT GAA CCA TTG AGA C | 97°1´, (97°0.75´, 56°0.75´, 72°1´) x 40 cycles, 72°4´ | 193 | *Treponema vincentii* (ex Brumpt) Simbert LA (ATCC 35580) |
| *Treponema pedis* | GGA GAT GAG GGA ATG CRT CTT CGA TG | CAA GAG TCG TAT TGC TAC GCT GAT ATA TC | 95°5´, (95°1´, 68°2´, 72°2´) x 40 cycles, 72°10´ | 424 | *Treponema pedis* T3552B Evans et al. 2009 (DSM 18691) |
| *Fusobacterium necrophorum* | GAG AGA GCT TTG CGT CC | TGG GCG CTG AGG TTC GAC | 94°5´, (94°1´, 60°0.5´, 72°2´) x 40 cycles, 72°10´ | 600 | *Fusobacterium necrophorum* subsp. *necrophorum* VPI 2891 [2358, JCM 3718] (Flugge) Moore and Holdeman (ATCC 25286) |
| *Dichelobacter nodosus* | GAA CGG TGC ATG GTT AAT AC | ACA TGA GTG TCA GTA TTG CC | 95°3´, (94°1´, 59°1´, 72°2´) x 35 cycles, 72°10´ | 312 | *Dichelobacter nodosus* VPI 2340 [11342] (Beveridge) Dewhirst et al. (ATCC 25549) |
| ⁰= degrees Celsius; ´ = minutes | | | | |  |


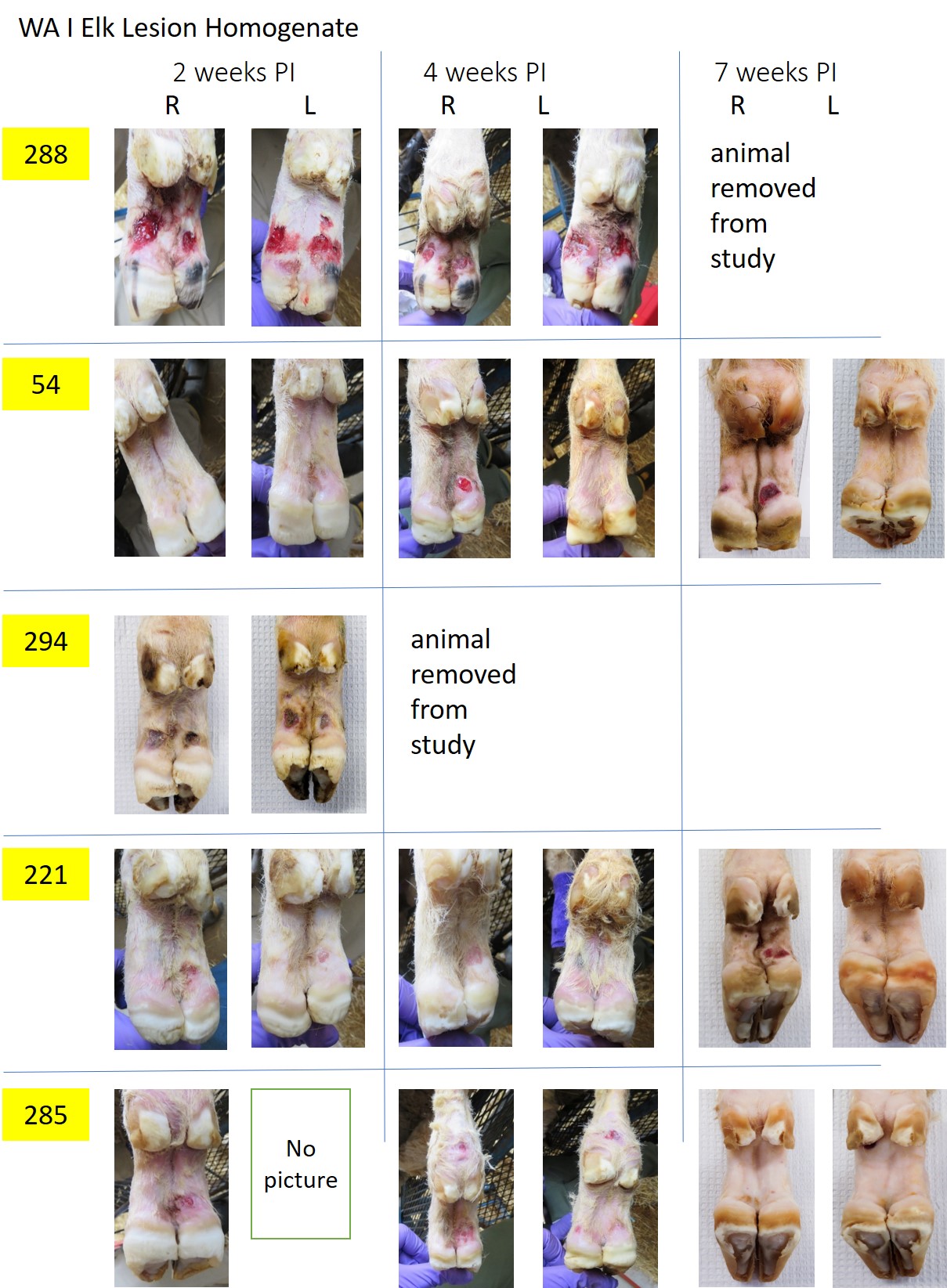
Figure S1: Lesion development at weeks 2, 4, and 7 for 5 sheep receiving WA I inoculum.


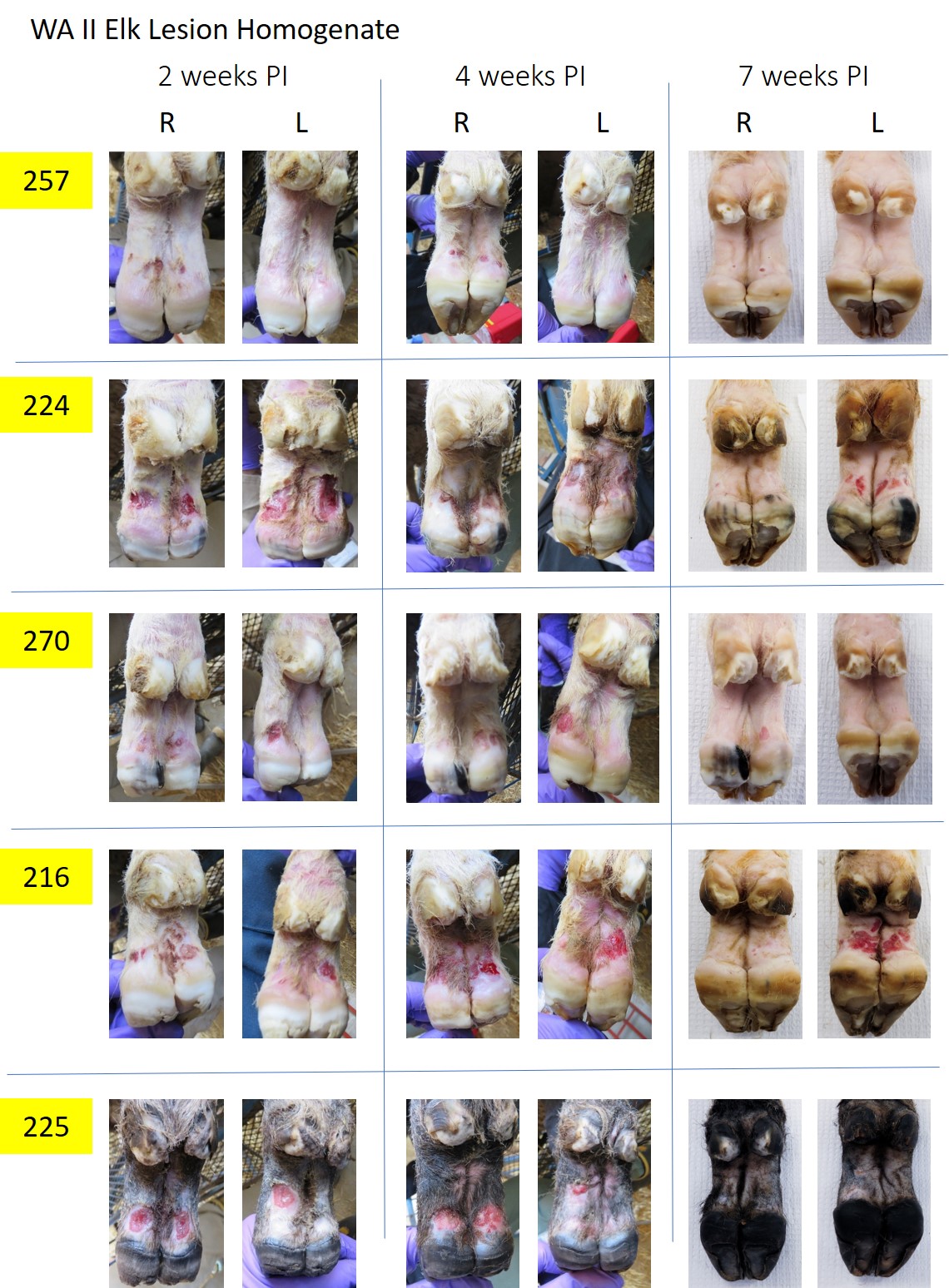
Figure S2: Lesion development at weeks 2, 4, and 7 for 5 sheep receiving WA II inoculum.


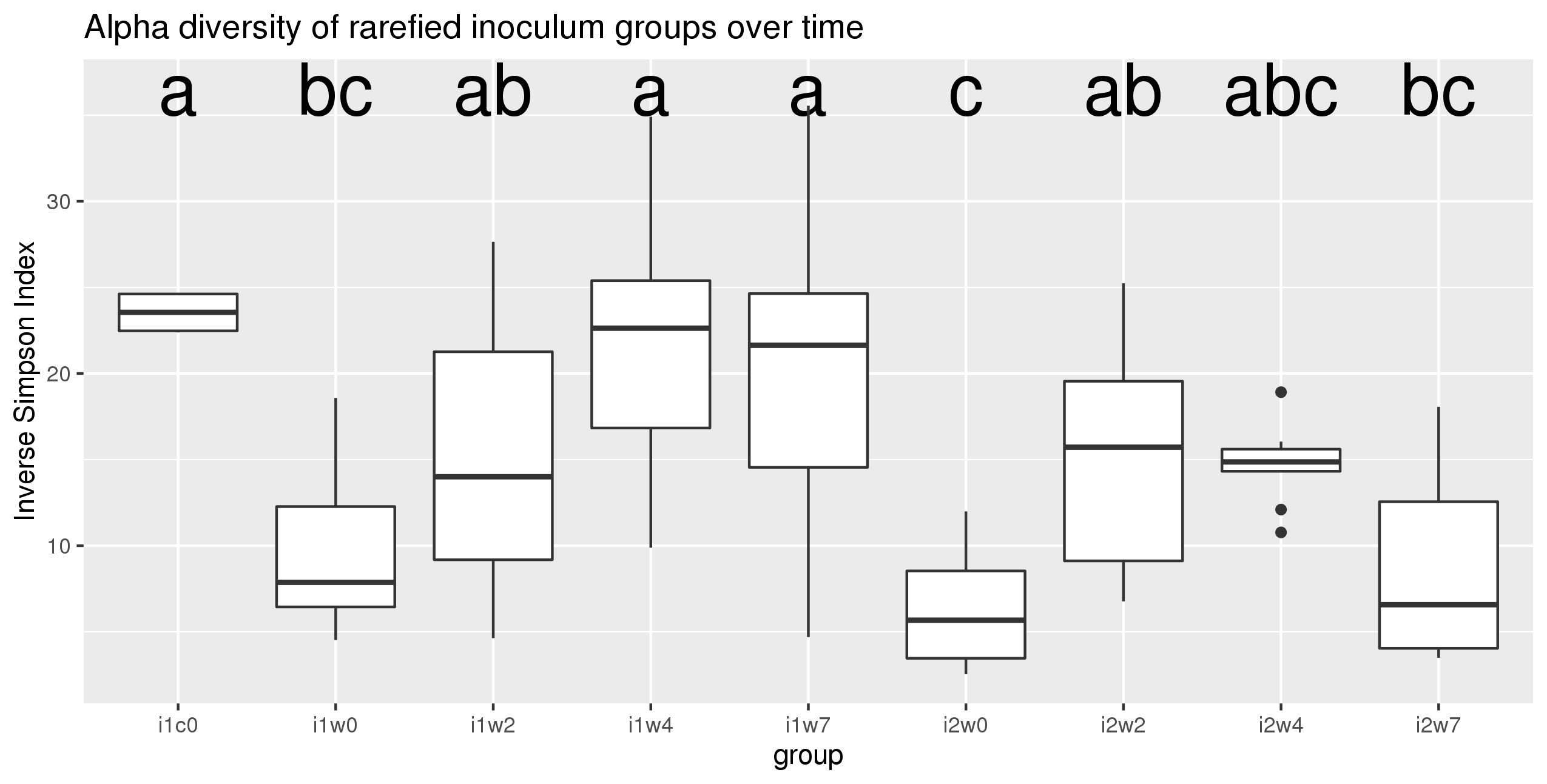


Figure S3: Inverse Simpson Alpha Diversity plot of relative diversity between inoculum (i1 TAHD or i2 mock) and timepoints. Bars with the same letter are not significant from each other (P<0.05) but are significantly different from bars with a different letter.


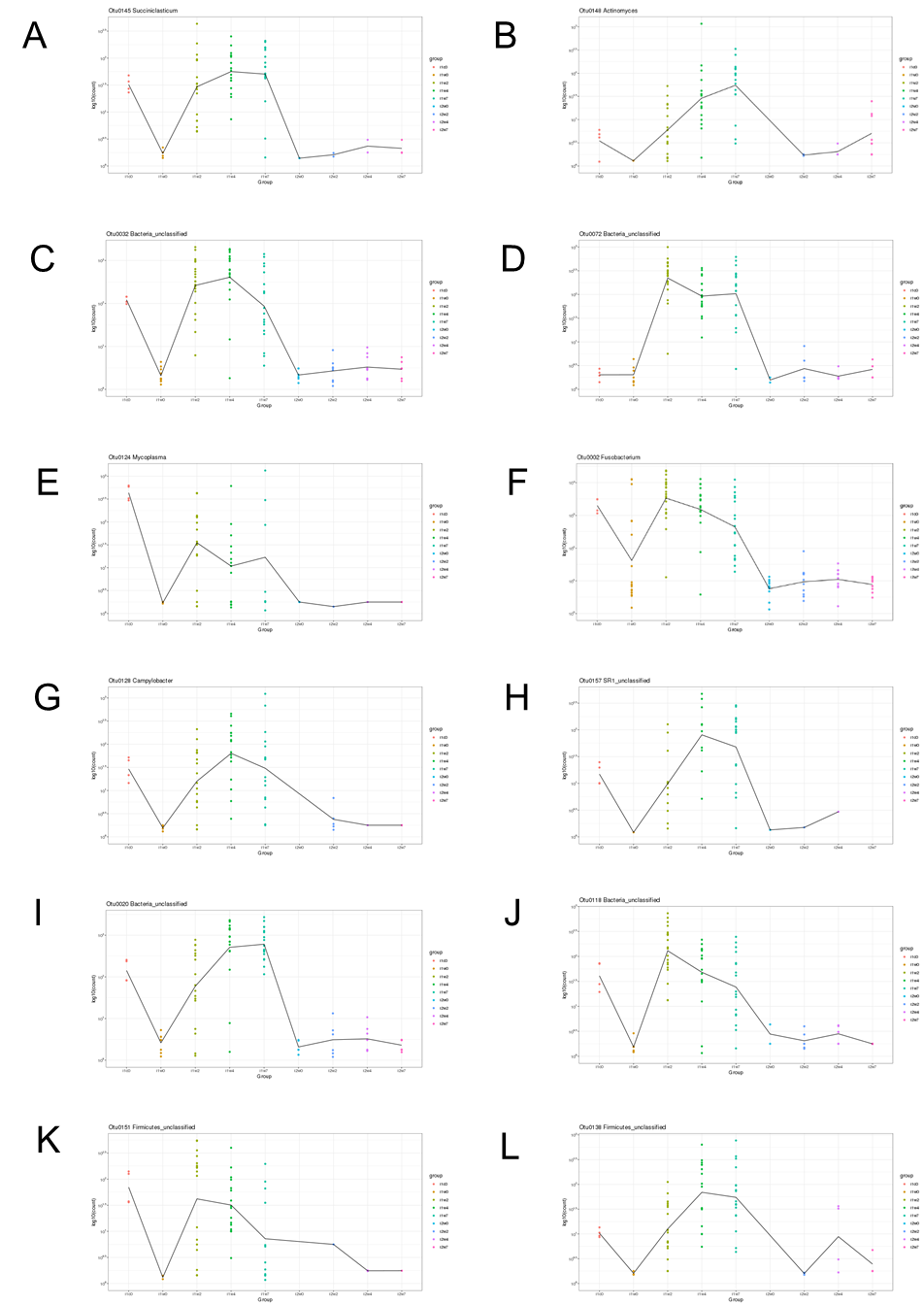


Figure S4: Relative abundance of bacterial phylotypes increased in inoculated samples over mock with mean counts greater than 20.


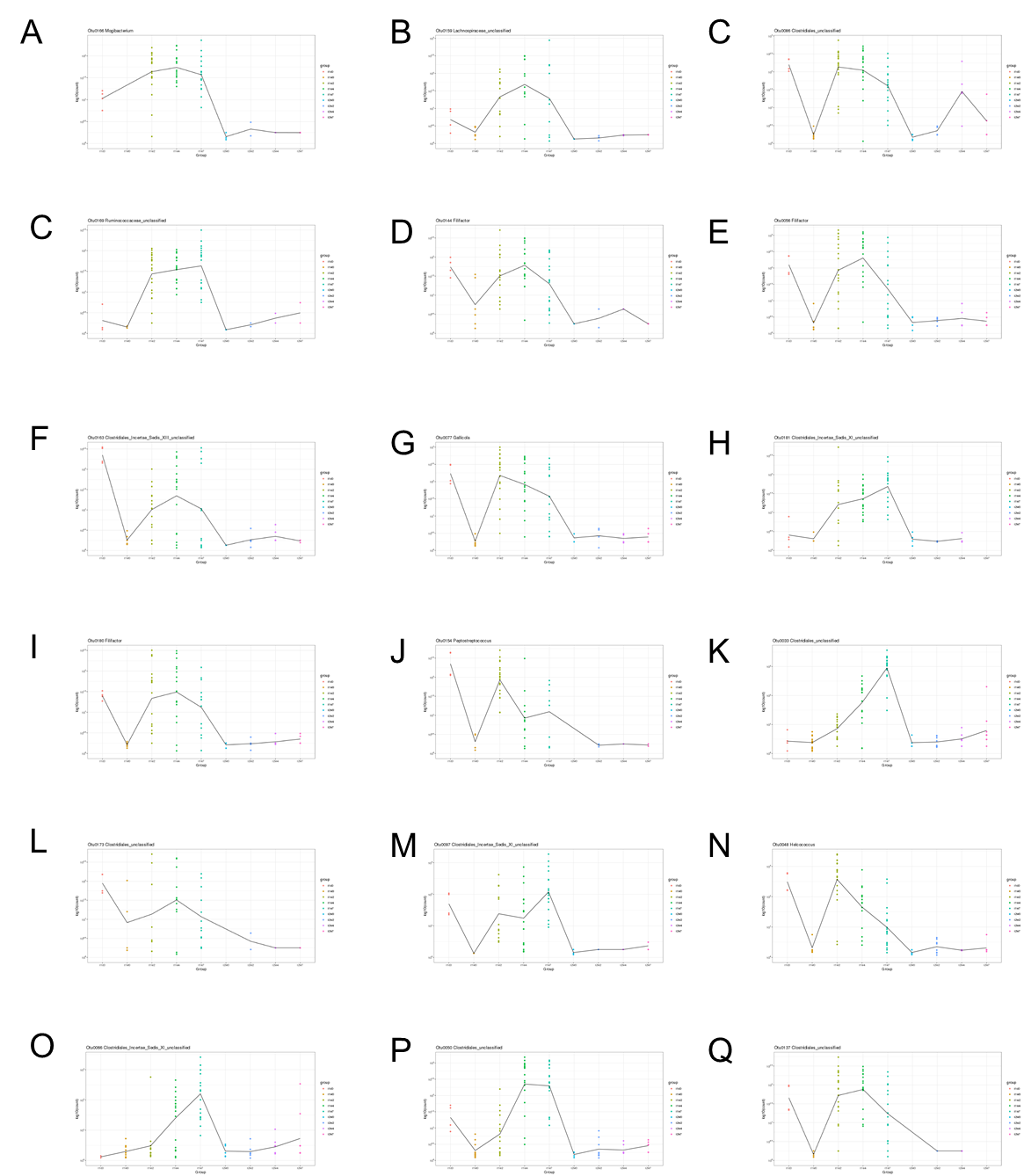


Figure S5: Relative abundance of Order Clostridiales phylotypes increased in inoculated samples over mock with mean counts greater than 20.


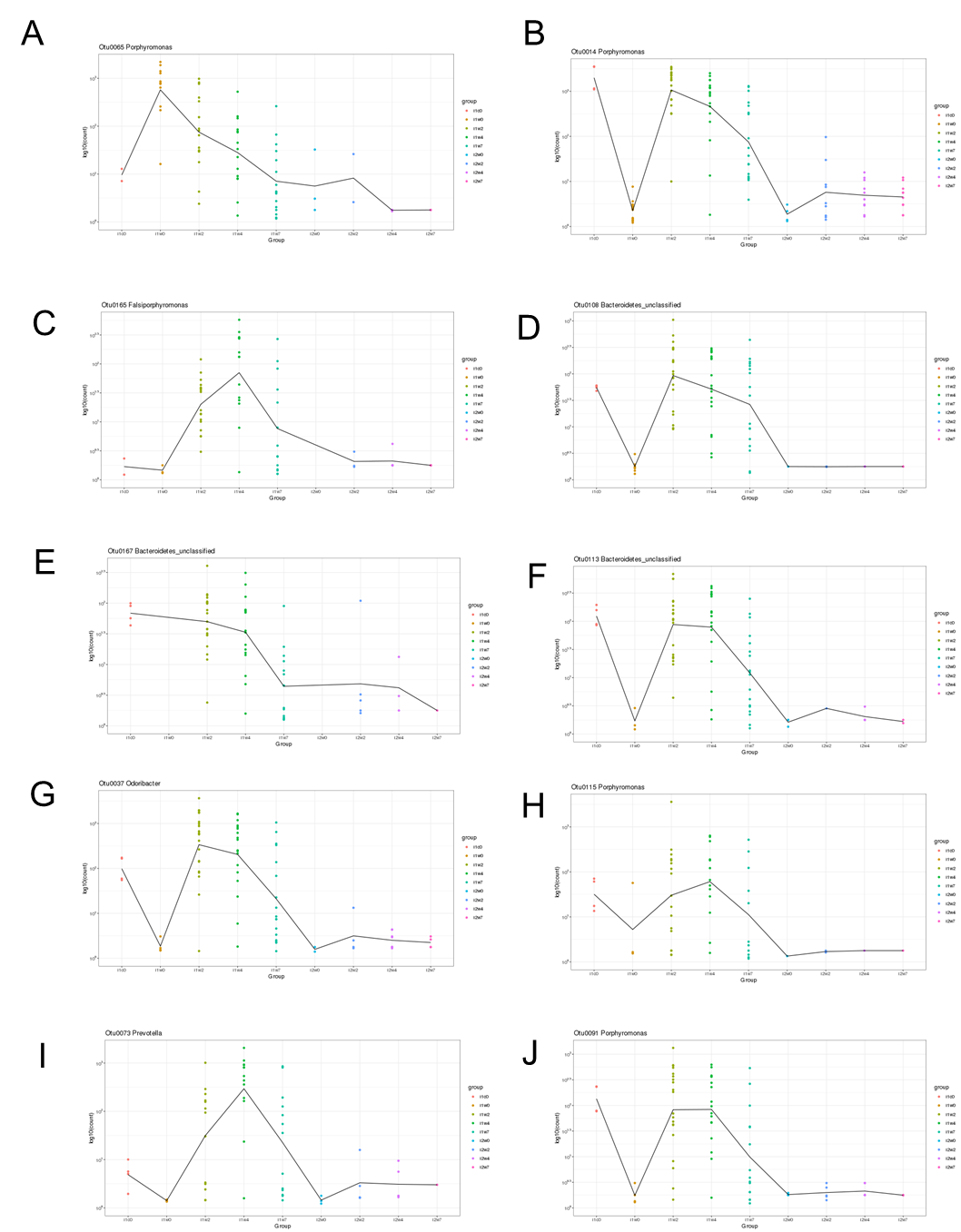


Figure S6: Relative abundance of Order Bacteroidales phylotypes increased in inoculated samples over mock with mean counts greater than 20.
